# More Than Pigments: The Potential of Astaxanthin and Bacterioruberin-Based Nanomedicines

**DOI:** 10.3390/pharmaceutics15071828

**Published:** 2023-06-26

**Authors:** Maria Jose Morilla, Kajal Ghosal, Eder Lilia Romero

**Affiliations:** 1 Nanomedicine Research and Development Centre (NARD), Science and Technology Department, National University of Quilmes, Roque Saenz Peña 352, Bernal 1876, Argentina; 2 Department of Pharmaceutical Technology, Jadavpur University, 188, Raja Subodh Chandra Mallick Rd., Jadavpur, Kolkata 700032, West Bengal, India; kajal.ghosal@gmail.com

**Keywords:** xanthophylls, inflammation, endocytosis, therapeutic, nanomedicines

## Abstract

Carotenoids are natural products regulated by the food sector, currently used as feed dyes and as antioxidants in dietary supplements and composing functional foods for human consumption. Of the nearly one thousand carotenoids described to date, only retinoids, derived from beta carotene, have the status of a drug and are regulated by the pharmaceutical sector. In this review, we address a novel field: the transformation of xanthophylls, particularly the highly marketed astaxanthin and the practically unknown bacterioruberin, in therapeutic agents by altering their pharmacokinetics, biodistribution, and pharmacodynamics through their formulation as nanomedicines. The antioxidant activity of xanthophylls is mediated by routes different from those of the classical oral anti-inflammatory drugs such as corticosteroids and non-steroidal anti-inflammatory drugs (NSAIDs): remarkably, xanthophylls lack therapeutic activity but also lack toxicity. Formulated as nanomedicines, xanthophylls gain therapeutic activity by mechanisms other than increased bioavailability. Loaded into ad hoc tailored nanoparticles to protect their structure throughout storage and during gastrointestinal transit or skin penetration, xanthophylls can be targeted and delivered to selected inflamed cell groups, achieving a massive intracellular concentration after endocytosis of small doses of formulation. Most first reports showing the activities of oral and topical anti-inflammatory xanthophyll-based nanomedicines against chronic diseases such as inflammatory bowel disease, psoriasis, atopic dermatitis, and dry eye disease emerged between 2020 and 2023. Here we discuss in detail their preclinical performance, mostly targeted vesicular and polymeric nanoparticles, on cellular models and in vivo. The results, although preliminary, are auspicious enough to speculate upon their potential use for oral or topical administration in the treatment of chronic inflammatory diseases.

## 1. Introduction

**Figure 1:**
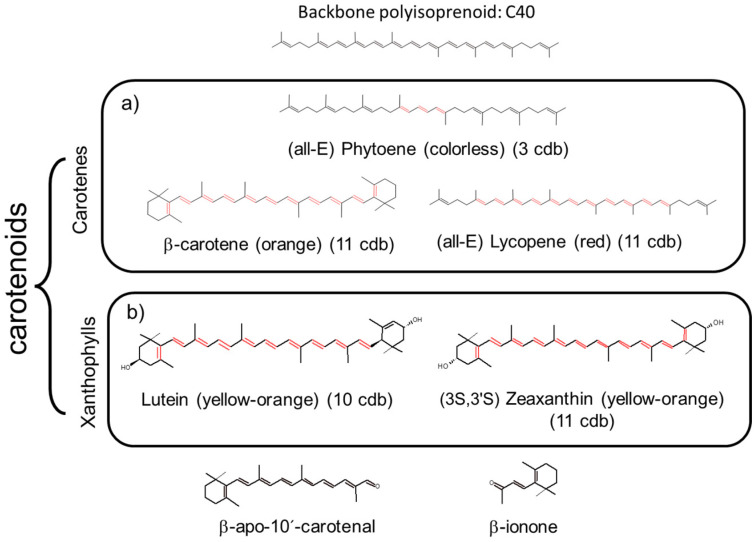
Carotenoid structures. (**a**) Structure of a representative carotenoid backbone with 11 cdb. (**b**) Representative structures of main C-40 carotenes and xanthophylls found in human tissues and <C-40 apocarotenoids.

Most of the intense antioxidant activity of dietary carotenoids comes from xanthophylls, a particular type of carotenoid. Since many inflammatory diseases are connected to oxidation, dietary xanthophylls are acknowledged to prevent chronic diseases’ damages, as a function of the polarity of their terminal ends and their ability to locate in specific positions in lipid bilayers of cell organoids [1]. Xanthophylls are potent reactive oxygen and nitrogen species (ROS and RNS) quenchers, free-radical scavengers, and chain-breaking antioxidants; display potent anti-inflammatory activity; and constitute potential therapeutic agents [1]. After being absorbed, carotenoids biodistribute in predictable patterns in animal tissues. Xanthophylls such as lutein, (*meso*)-zeaxanthin, and zeaxanthin are highly concentrated in the macula lutea, where they prevent photochemical damage, delaying the development of age-related macular degeneration (a very common vision disorder in older adults) and glaucoma [2,3]. Their esterified forms act as UV absorbers and quenchers of singlet oxygen in the skin surface and subcutaneous tissue [4]. *β*-cryptoxanthin, lutein, and zeaxanthin (Figure 1) are found in the brain and exert beneficial effects on cognition [5,6]. Lycopene is the only carotene that accumulates in the prostate [7].

However, pathological or damaged tissues do not necessarily match the natural distribution pattern of carotenoids. If xanthophyll’s activities were exerted on tissues other than those accessed naturally, their activities could be magnified and employed as preventive agents and as treatments against diseases. To that aim, they need to be formulated as nanomedicines. Nanomedicines not only protect the labile structure of different loaded molecules, natural products included, against degradative environments such as the gastrointestinal tract but also allow the control of their pharmacokinetics (PK), biodistribution (BD), and pharmacodynamics (PD), magnifying their activity. Since nanomedicines can be administered by routes other than oral, new tissues, cells, and intracellular compartments could be targeted, resulting in new molecular entities of natural origin, of potentially very high added value.

Hence, this review is aimed to show the preclinical performance of astaxanthin (AST) and bacterioruberin (BR)-based nanomedicines, to treat inflammatory diseases. After a general introduction to the structural and functional nature of carotenoids, the main structural, biophysical, and biochemical aspects of AST and BR are summarized, including the reasons for the interest raised by a rare carotenoid such as BR, and clarifying the terminology responsible for puzzling differences between foods and nanomedicines. On those bases, the available articles (excluding in silico or in the absence of cells, disease, or animal model reports) on the performance of each nanomedical formulation are thoroughly described. The impact of these preclinical findings and further perspectives for the nanomedical field are finally discussed.

## 2. Materials and Methods

Bibliographic searches were carried out in Science Direct and PubMed of original publication in English, excluding reviews, with the following research terms: astaxanthin/bacterioruberin and (nanomedicines, nanoparticles, polymeric nanoparticles, solid lipid nanoparticles, micelles, or liposomes). Publications that merely focused on technological developments without any biological or biorelevant assay were excluded. For this, the following keywords were included in the search: inflammatory bowel diseases, psoriasis, atopic dermatitis, dry eye, arthritis, and inflammation. The bibliometric analysis showed the citations of reports including the word “astaxanthin” raised from 86 in 2000 to 1235 in 2022, accounting a total of 9826 up to 5/2023, while between 2000 and 2023, the analysis indicated only 359 citations of reports including the word “bacterioruberin” up to 5/2023, almost 30 times fewer papers than those on AST. The research yielded only 18 scientific articles, the performance of which will be described.

## 3. Carotenoids: Structure and Source, Dietary Effects in Animals, and Human Consumption

Carotenoids are tetraterpenoid pigments, sharing a common structure: a long polyene chain with 8–13 conjugated double bonds (cdb) (Figure 1) that is the chromophore responsible for its coloration/pigmentation properties (absorption of light in the visible range (400–550 nm) of the electromagnetic spectrum) [8,9].

As the number of cdb is increased, carotenoids range from colorless to deep red [10]. Carotenoids are widely distributed in nature. Currently, 722 organisms (mainly photosynthetic: higher plants, phytoplankton and phototrophic bacteria, and micro- and macro-algae) and, to a lesser extent, non-photosynthetic organisms (yeast, fungi, and archaea) have been described as de novo synthesizers of the 1204 natural carotenoids outlined in the Carotenoid Database [11,12,13].

Most carotenoids possess a C-40 skeleton (1121). There are about 120 degradation products known as apo-carotenoids (< C-40) including 33 diapo-carotenoids (C-30) [14], which are responsible for aroma, color, and phytohormone production (abscisic acid and strigolactones) in plants [15,16,17] as well as attractants for other organisms, such as insect pollinators and seed-dispersing herbivores [18]. There are also known 13 C-45 and 37 C-50 higher carotenoids, 13 exclusively found in some species of archaea [19] that stabilize the structure of membranes in addition to their basic biochemical functions.

In plants and microalgae, carotenoids are stored in plastids (tilakoids), where their most prominent functions are oxygenic photosynthesis (light harvesting) and photoprotection (detoxification against photodynamic destruction generated during photosynthesis).

The polyene backbone of the acyclic hydrocarbon β-carotene (C_40_H_56_), modified by either hydrogenation, dehydrogenation, cyclization, oxidation, or any combination of these processes, is common to the two types of carotenoids: carotenes (hydrocarbons, molecules lacking oxygen atoms, 50 kinds found in nature) such as *α*-carotene, *β*-carotene, *β*,*ψ*-carotene (*γ*-carotene), and lycopene [20], and xanthophylls (molecules having oxygen atoms, namely, hydroxy, carbonyl, aldehyde, carboxylic, epoxide, and furanoxide groups) such as *β*-cryptoxanthin, lutein, zeaxanthin, astaxanthin, fucoxanthin, and peridinin (Figure 1). Some natural xanthophylls are found as fatty acid esters, glycosides, sulfates, and protein complexes, showing higher diversity than carotenes. About 800 kinds of xanthophylls have been reported in nature up until 2018 [13].

Animals cannot synthesize carotenoids but selectively absorb and modify specific carotenoids derived from dietary sources [21]. Dietary carotenoids (nearly 40, with lycopene, *α*-carotene and *β*-carotene, lutein, zeaxanthin, and *β*-cryptoxanthin being the main six found in the blood) are the unique source of vitamin A, a potent gene regulator controlling the expression of about 700 genes. Only the unsubstituted β-ionone ring carotenes, such as β-carotene, the most abundant dietary carotenoid in human tissues, are precursors of different forms of vitamin A: vitamin A1 [retinol], vitamin A2 [3,4-didehydroretinol], and vitamin A1 aldehyde [retinal], needed for animal vision and signal transduction [retinal-bacteriorhodopsin] in halophile microorganisms and retinoids [22].

In animals such as birds, where they are used in ornamental displays, and fishes, dietary carotenoids are also an important indication of a satisfactory nutritional condition and fitness and are used to increase sexual attractiveness. In hens [23,24] and salmons, carotenoid ingestion increases fecundity, growth rate, egg yolk volume and quantity, and intensity of flesh color and strengthens immune responses.

The world consumption of carotenoids is measured in thousands of tons per year added to food or beverages and gives rise to a global carotenoid market characterized by its dynamism and intense competitive conditions. Between 2019 and 2026 the global market of carotenoids is expected to grow from USD 1.5 to 2 billion at a compound annual growth rate (CAGR) of 4.2%. The carotenoid market is pulled by the trend to use natural colorants, their applications in human health, and the development of new extraction techniques. Consumers across the world are demanding clean-label products due to increasing concern over the use of synthetic ingredients in food. It is estimated that in the next years the demand for natural products (foods, beverages, and cosmetics) in replacement of synthetic ones will grow [25]. North America and Europe have the largest carotenoid market, owing to the growing demand for clean-label foods and health awareness [26]. Europe is projected to account for the largest share in the natural carotenoid market, due to the presence of leading manufacturers, and the surge in demand for molecules such as lutein (driven by its use in age-related eye disease/cataracts, macular degeneration) [27], lycopene, and β-carotene (to reduce the risk of chronic diseases such as diabetes, cancer, etc.) by ageing consumers that preferer opting for natural clean label products [28]. The feed segment is predicted to account for the largest market share. Between 2022 and 2028, the feed carotenoid market is projected to register a CAGR of 2.2–3.6% and is predicted to surpass a revenue of USD 2.75 billion. In the animal food production sector, aquaculture is the fastest growing segment worldwide [29] because of its use as a coloring of fish meat in the Asia–Pacific area since the color is perceived as a healthy property, stimulating the reason for its high consumption. In such context, the Asia–Pacific area is an emerging market, growing at the highest CAGR over 2018–2028 [30].

Currently, carotenoids are mainly produced by chemical synthesis since the yield of natural sources is still low (<10% of carotenoids per dry weight). Synthetic carotenoids are produced faster and cheaper than natural ones, but they are less effective in terms of their health-promoting properties and are hence less valuable and desirable as a product [31].

The modern public perception considers natural products as less noxious than those chemically synthesized. A growing amount of research proves that carotenoid consumption would be beneficial not only for animal welfare but also to reduce the incidence of human chronic inflammatory diseases [32]. Taken together, these factors picture a complex present aimed to favor the rise of companies selling carotenoids produced and extracted from natural sources, encompassing the circular economy and genetically modified organisms [33].

## 4. Antioxidant and Anti-Inflammatory Activity of Xanthophylls

**Figure 2:**
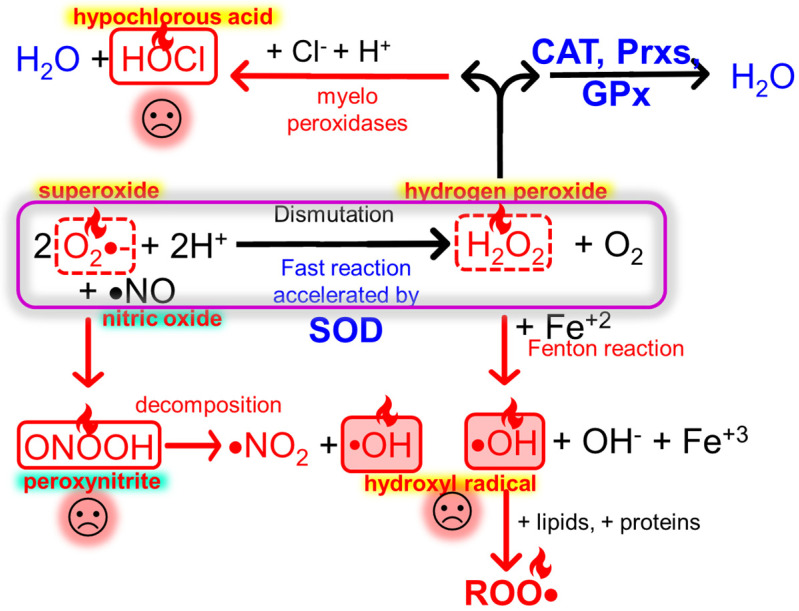
Mechanisms of ROS and RNS generation and endogenous antioxidant systems in animals. Reactive species (ROS (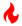) and 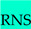) (highly reactive (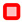); highly oxidant (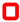); less reactive (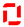)) are generated both by endogenous (mitochondrial respiratory chain, prostaglandin synthesis, and phagocytosis) and exogenous (exposure to environmental pollutions, heavy metals, certain drugs, organic solvents, alcohol, and radiations) stimuli. By direct reactions, •OH, ONOO-, and HOCl oxidate proteins and generate nucleic acid fragmentation, mutagenic lesions, and peroxidase lipids, which lead to malfunction and cellular death (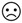). To defend against oxidative damage, organisms have endogenous antioxidant systems, which include (i) antioxidant enzymes (superoxide dismutase (SOD), catalase (CAT), glutathione peroxidases (GPx), and peroxiredoxins (Prxs) [remove H_2_O_2_]), (ii) substrates of antioxidant enzymes (glutathione (GSH), thioredoxin (Trx), and NADPH), and (iii) low-molecular-mass antioxidants (bilirubin, albumin, uric acid, α-lipoic acid, melatonin, and coenzyme Q10). In addition, organisms employed exogenous antioxidants only present in microbial or plant cell such as vitamin C, vitamin E, carotenoids, polyphenols, flavonoids, and metals Se, Cu, Zn, and Mn (co-factors of enzymes). The effectivity of the antioxidant system depends on the capacity to maintain redox homeostasis controlling the generation and elimination of O_2_•−, H_2_O_2_, and •NO at levels that limit the production of •OH and ONOO- [37]. The only effective strategy to reduce the damage induced by •OH is to avoid its formation by preventing O_2_•− formation and eliminating O_2_•− and H_2_O_2_. Remotion of O_2_•− prevents the formation of ONOO−, while remotion of H_2_O_2_ prevents the formation of •OH and HOX [X: halogen] [35].

Oxidative stress (OS) occurs when an excessive generation of oxidative species overcomes the antioxidant systems; in such a situation, a disruption of redox signaling and molecular damage takes place [34]. In normal situations, the production and elimination of ROS and RNS are precisely controlled by endogenous and exogenous antioxidant systems (Figure 2). Elevated levels of reactive species during prolonged periods cause structural defects in mitochondrial DNA and genetic expression and enzyme and plasma membrane alterations, which lead to the development and progression of several diseases. The OS is the primary cause of radiation-induced lung injury and atherosclerosis and contributes to the development of chronic obstructive pulmonary disease (COPD), type 2 diabetes mellitus, idiopathic pulmonary fibrosis, hypertension, ischemia–reperfusion injury, Alzheimer’s disease, and cancer [35,36].

OS and inflammation are closely related and are linked to pathological events. Chronic inflammation and OS coexistence have been reported in many chronic affections such as diabetes, cardiovascular, renal and neurodegenerative diseases, inflammatory bowel disease (IBD), arthritis, and psoriasis. Inflammatory cells release cytokines that exacerbate OS, whereas ROS and RNS trigger the intracellular signaling cascade that increases the expression of inflammatory genes [37,38]. The signaling routes NF-κβ (Nuclear factor kappa-light-chain-enhancer of activated B cells) and Nrf2 (Nuclear factor erythroid-related factor 2) co-regulate a wide spectrum of biological functions in response to inflammation and OS [39].

NF-κβ controls the expression of cytokines and pro-inflammatory chemokines, growth factors, immuno-receptors, cell adhesion molecules, and anti-apoptotic proteins. Nrf2, on the other hand, regulates the redox homeostasis and controls the expression of proteins involved in the antioxidant defense, the removal of oxidized proteins by the proteasome, and detoxification, apoptosis, autophagy, DNA reparation, and inflammation [40,41]. There is a complex interrelation between NF-κβ and Nrf2 pathways, whose complex regulation is not fully elucidated yet but that can be roughly described as a mutual inhibition [42].

The more effective strategies to treat chronic diseases that involve OS and inflammation are those that reduce the production of oxidants and at the same time inhibit the downstream signals that result in inflammation and cell death, increasing the pool of antioxidant enzymes and their substrates [35].

**Figure 3:**
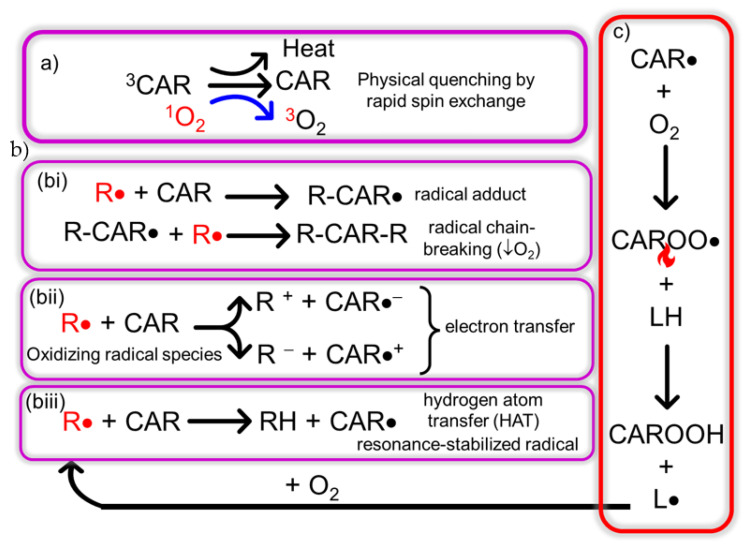
Carotenoids as ROS quenchers. Antioxidant mechanisms common to all carotenoids. (**a**) Singlet oxygen (^1^O_2_) physical quenching, (**b**) free radicals quenching (**bi**) radical adduct mechanism; (**bii**) electron transfer mechanism; (**biii**) hidrogen atom transfer mechanism; and (**c**) carotenoids oxidation mechanism and generation of pro-oxidative species at high O_2_ levels.

The antioxidant activity of carotenoids is exerted at distinct levels. They are efficient singlet oxygen ^1^O_2_ quenchers, converting the ^1^O_2_ produced from chlorophyll triplet states during photosynthesis to ^3^O_2_ via physical quenching by a rapid spin exchange while the carotenoid triplet decays to the ground state [43] and by chemical quenching by β-carotene leading to β-carotene endoperoxide [44] (Figure 3). Carotenoids are also scavengers of free radicals by different chemical mechanisms [45].

The antioxidant activity of carotenoids is intensified from colorless carotenes with short polyene chains (phytoene and phytofluene), to the intermediate-length carotenes such as beta carotene, to become maximal for long-chain, polar ends carotenoids [46]. Nonaene chromophore or longer conjugated system carotenoids are also photoprotectors.

We have selected AST and BR as examples of xanthophylls extremely disparate in terms of popularity: if the second is practically unknown, its source and properties may render it as competitive for the pharmaceutical market than the former one. In the next section, their structural and functional characteristics (summarized in Table 1) will be reviewed.

## 5. The Highly Marketable Astaxanthin

AST is the xanthophyll that attracts the most commercial and academic interest. In 2023, the AST segment is expected to account for the largest share, dominating the carotenoids market [25]. AST is a dark red C-40 ketocarotenoid (3,3′ -dihydroxy-β, β-carotene-4,4′ -dione), with 13 cdb, whose activity depends on its source and extraction method. The highest-quality AST is obtained from primary biological sources such as the microalgae *Haematococcus pluvialis* [84,85]; these and other alternative biological sources such as Gram-negative bacteria, fish, yeast, and krill are being permanently explored and improved [33,86,87,88]. AST may also be chemically synthesized from petroleum distillate [89]. Synthetic AST displays lower antioxidant activity since it consists of a mixture of isomers (Table 1) and is not recommended for human consumption due to concerns regarding food safety [90]. The only FDA-approved AST for human consumption is the AST extracted from *H. pluvialis* [91].

AST can neither be converted into vitamin A nor synthesized de novo by animals; therefore, it must be consumed in the diet [92]. Through feeding, AST is present in egg yolks and feathers from flamingos, canaries, and chickens, and it confers salmon, shrimp, and lobster shells their distinctive color and plays key metabolic effects (Table 1) [49]. In human consumption, it is employed as food supplements and cosmetic ingredients.

**Figure 4:**
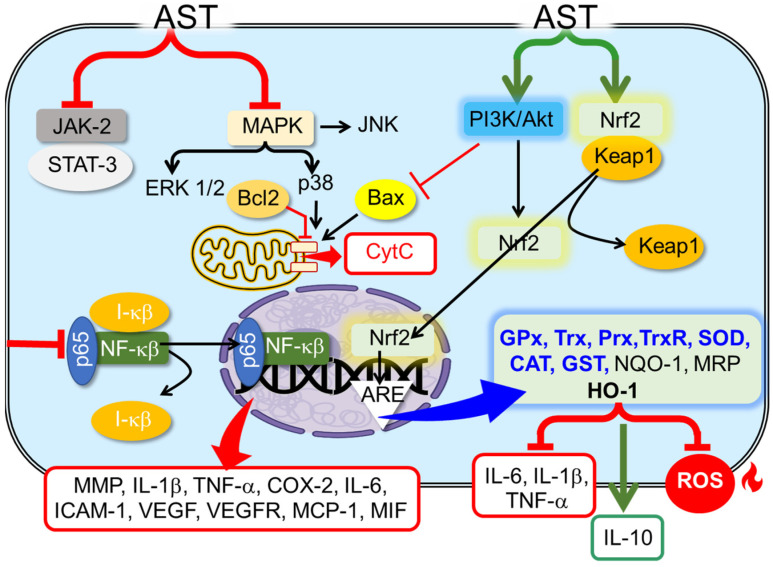
Antioxidant activity of AST and signaling pathways. AST activates the Nrf2 pathway, which regulates the expression of 250 genes in the promoter region known as Antioxidant Response Element (ARE). Nrf2 is regulated by several pathways at different levels; one of these, Kelch-like ECH-associated protein 1 (Keap1), is a cytoplasmatic repressor that facilitates the degradation of Nrf2 through the proteasome. In the presence of electrophilic molecules (reactive species and oxidants), Keap1 is alkylated and loses its ability to cause degradation of Nrf2, which results in the nucleus accumulation of Nrf2. In the nucleus, Nrf2 forms dimers with small musculoaponeurotic fibrosarcoma oncogene homologue (sMAF, Nrf2–sMAF) proteins and induces ARE-containing genes [42,96]. These genes can be divided into three categories: cellular antioxidants, such as Gpx, Trx, Prx, and thioredoxin reductase (TrxR); phase II detoxificant enzymes, such as heme oxygenase-1 (HO-1), glutathione S-transferases (GST), dehydrogenase quinone 1 (NQO1), CAT, and SOD; and transporters, such as multidrug resistance-associated protein (MRP) [97]. Nrf2-Keap1 also attenuates IκBκ phosphorylation in the canonical NF-κB activation pathway thereby reducing the nuclear accumulation of NF-κB [98]. On the other hand, an increase in the expression of HO-1, activated by Nrf2, leads to the inhibition of NF-κB and attenuation of inflammatory reactions. Nrf2 can also suppress the transcriptional activation of inflammatory genes (e.g., IL-6 and IL-1β) without binding to ARE sequence, by direct binding to promoter regions and inhibiting the recruitment of RNA Pol II [99]. AST also promotes PI3K/Akt. PI3K and Akt trigger several substrates and downstream effectors such as mTOR, GSK-3β, HIF-1α, Bcl-2, Bad, FoxO, and a number of other cellular proteins associated with metabolism, cell survival/apoptosis, differentiation, and proliferation [77]. AST prevents the production of pro-inflammatory cytokines by suppressing the phosphorylation of p38, ERK, c-Jun N-terminal kinase (JNK), and mitogen-activated protein kinases (MAPKs) and also inhibits NF-κB activation, which leads to the inhibition of downstream molecules [100]. High intracellular ROS levels can induce apoptosis by activation of the proapoptotic proteins Bax, p21, and p27, among others, and a decrease in the antiapoptotic Bcl-2 and Bcl-xL. AST inhibits the translocation of the proapoptotic Bax and Bad proteins to the mitochondria and that of Bcl-2 from the mitochondria to the cytosol, as well as the release of cytochrome C into the cytoplasm. This process attenuates MMPs expression, thereby inhibiting the release of proapoptotic molecules that trigger caspase activation and contribute to apoptosis [101].

AST is known to modulate different signaling pathways (Figure 4) and mitigate different chronic diseases in both animal models and clinical trials (Table 1). Natural AST displays anti-inflammatory and antioxidant activity in vivo [72,79,93,94,95], prevents cardiovascular diseases, is neuroprotector, improves visual acuity and retinal blood flow [82], decreases the sign of aging on the skin [83], and promotes immune responses [61,89].

The effects of AST are related to its ability to associate in high quantities with the membrane of cellular organoids, which in turn depends on its source, which regulates the isomeric variety and esterification. The extraction method and storage condition may modify these features and must also be reported, to rigorously analyze the properties of AST. For in vitro studies, where bioaccessibility and bioavailability are not relevant, it is indispensable to know at least its isomeric form.

**Figure 5:**
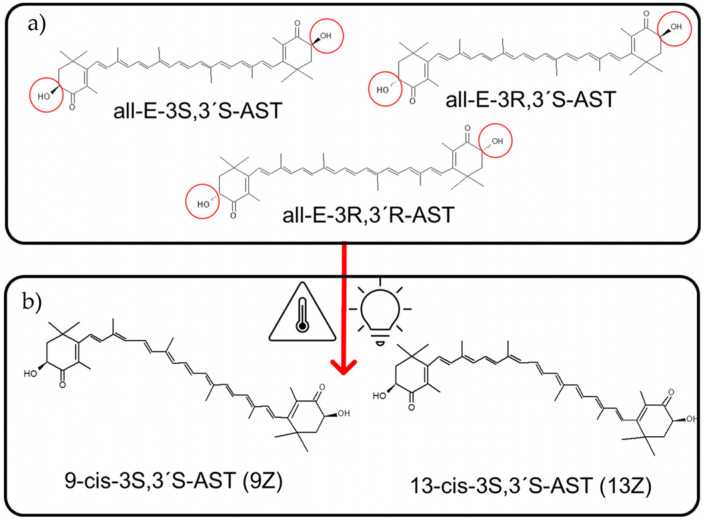
Representative structures of different geometric and optical isomers of AST. The backbone in the all *E*(all *trans*)-configurations shown in (**a**) is lineal; the two residual parts of the molecule lie on opposite sides of the plane. In the Z-configurations shown in (**b**), instead, the introduction of *cis* bond bends the molecule so as the extremes lie at the same side of the plane. Heat and light may induce conformational changes.

The bioavailability and activity of AST, for instance, depend on the type of geometric (*trans* or *cis*) isomer (Figure 5). Most carotenoids found in nature are predominantly all-*trans* isomers (*trans* isomers are known as E-isomers). *Cis*-isomers (known as Z-isomers) instead are less thermodynamically stable. During storage, the transformation of Z isomers (amorphous oily) into E (more crystalline and insoluble) may occur. All-E AST, however, may be readily isomerized to *cis*-*trans* mixtures, especially 9-*cis* and 13-*cis*. Light irradiation, heat, and catalytic treatments, such as natural catalysts, namely, isothiocyanates and polysulfides, naturally present in mustard, onion, and garlic, may cause the (Z)-isomerization of all-*E* backbone and have been utilized in the (Z)-isomerization of lycopene, β-carotene, and AST [102]. The absence of these structural data detracts from the accuracy of AST’s activity reports.

AST is extracted from *H. pluvialis* as pure all-E-(3S,3′S) stereoisomer. Given the length of AST hydrocarbon backbone, about 30 Å [103] (near the thickness of lipid bilayers approximately 25–32 Å) [104], the linear all-E-(3S,3′S) isomer is the only capable of partitioning perpendicularly to the membrane plane, a position stabilized by the C4 and C4’ ketone group from the terminal rings. Such orientation interferes with the propagation of free radicals in the hydrophobic core and quenches radicals generated at the surface of membranes; its antioxidant activity thus is enhanced by providing protection throughout the entire depth of membranes [105,106]. Its chemical structure and position together critically influence significant aspects of cellular functionalism. The other optical isomers lack the extensive metabolic effects of 3S,3′S and merely lend a colorant and antioxidant function. Synthetic AST, for example, is a mixture of isomers, very toxic to young fish because it interferes with ROS signaling functions [107].

Mitochondria are organoids exposed to high partial oxygen pressure and are the main sites of the production of ROS [36]. It has been observed that AST is a mitochondria-targeted antioxidant [79], which thanks to perpendicular insertion into membranes prevents dysfunctions related to the mitochondrial ROS generation [108,109] by protecting its membrane lipids from peroxidation [106,110]. AST is acknowledged as an increaser of ATP production, mitochondrial number, and respiratory chain complex activity [111,112].

The existence or not of esterification must also be reported in in vivo studies of AST [107,113]. Esterification plays a critical role in the bioavailability and subsequent activity of AST. The monoesters and diesters of AST extracted from *H. pluvialis* present the higher bioavailability, which is highest for shorter and unsaturated esters [114], resulting in more stability in front to oxidation, providing higher antioxidant activity both in vitro and in vivo [115]. Esterified AST is more effective to protect mitochondria from oxidative damage during exercise than free AST [116,117] and was found to provide higher antitumoral protection [86].

**Figure 6:**
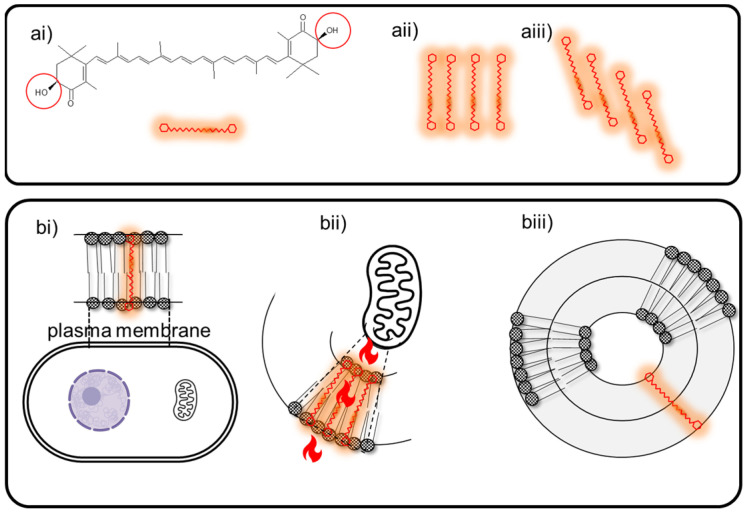
Representative insertion of 3S,3S’AST in lipid bilayers. (**ai**) 3S,3S’AST monomer (hydrophilic OH groups in red), (**aii**) H aggregate, and (**aiii**) J aggregate. (**bi**) The angles on the C-3 and C-3’from 3S,3S’AST favor this isomer taking up residence in the cell plasma membrane since these are angled to anchor the hydrophilic elements (the rings) of the AST in the hydrophilic lipid heads and the hydrophobic element of the AST (the conjugated backbone) with the lipid tails. In this way, AST can quench ROS produced both in the core of the bilayer (reducing lipid peroxidation) and in the hydrophilic lipid’s heads. (**bii**) AST in mitochondrial membrane. (**biii**) AST in liposomal bilayer.

In thylakoids and in lipid bilayers, xanthophylls are found in aggregated forms [118]. In liposomes, AST is partitioned as monomers, and as its proportion increases, it forms H (molecules tightly located in face-to-face form) or J aggregates (molecules loosely located in head-to-tail form) [113,119]. Monomers and aggregates display different optical features and physical and chemical properties including antioxidant activity [120,121], each affecting the physical and dynamic properties of lipid bilayers [122]. H aggregates provide rigidity, compactness, and extra protection to the lipid bilayer, while J aggregates could greatly improve the storage stability of liposomes (Figure 6). Carotenoid aggregation has been reported to reduce their ability to neutralize singlet oxygen [123]. On the contrary, other authors have found that the H aggregates display higher intracellular antioxidant activity than the monomeric form, as measured by DPPH and hydroxyl radical scavenger techniques [120,124,125]. Instead, carotenes such as lycopene and β-carotene, because of their hydrophobic nature, are inserted parallel to the surface of the bilayer, disordering the non-polar interior [122,126]; consequently, their ability to interact with ROS in the hydrophilic environment is much lower than that of xanthophylls. In addition, in the presence of certain metal cations such as Fe^2+^ and high partial pressures of oxygen, carotenes exhibit pro-oxidant activity [127]. So far, no pro-oxidant of AST activity has been recorded [122].

## 6. Bacterioruberin, a Xanthophyll Hidden in the Salt

BR, on the other hand, is not produced synthetically and is considered a “rare” carotenoid [19]. Unlike AST, BR is virtually unknown to the academic community. The biophysics and therapeutic properties of BR are almost unexplored (Table 1). However, the experimental evidence collected so far suggests that BR could offer activity comparable or superior to that of AST.

BR is a xanthophyll mainly produced by archaeal halophilic microorganisms [128,129]. Halophilic microorganisms (including marine and those living in solar salt flats) grow optimally in 2.5–5.2 M NaCl, can be found in places with salt concentrations as high as halite saturation [130], and can survive extreme desiccation, starvation, and radiation, seemingly for millions of years [131].

**Figure 7:**
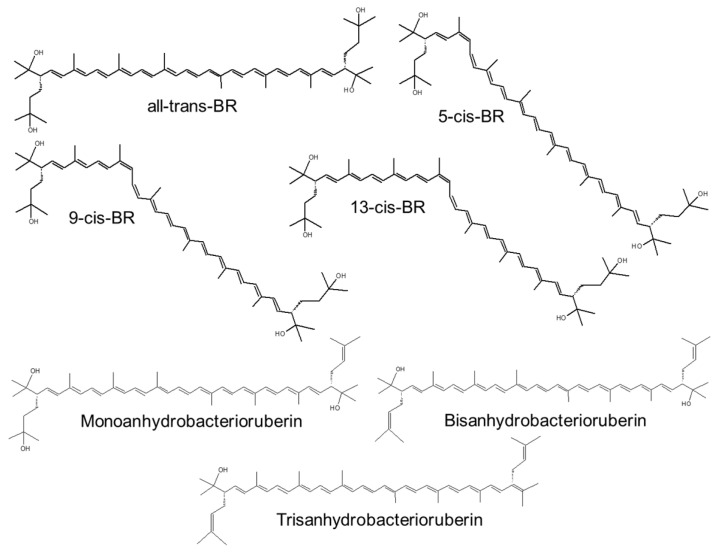
BR structure, isomers, and derivatives. BR presents several geometric isomers; all-trans-BR, the most abundant, represents between 28% and 65% of the total carotenoid content. The cis-isomers (5-cis-BR, 9-cis-BR, and 13-cis-BR) are found in variable amounts (4.6–19.2%, 5.8–12.8%, and 2.6–14.8%, respectively) depending on the *Haloarchaea* species [66,68,136]. Double cis-BR isomers (5-cis-26-cis-BR and 9-cis-26-cis, 3.7–7.2%) are also found in *Haloarchaea*. The very few exhaustive studies describing the composition of *Haloarchaea* extracts and differences in the analytical systems used and extraction methods, including the solvents, could explain the different number of BR isomers and derivatives found in *Haloarchaea*. To this must be added that the nutritional conditions and other culture conditions influence the BR isomers produced. For example, in *Hfx. Mediterranei*, increasing concentrations of carbon led to higher percentages of all-trans-BR, 5-cis-BR, and the double isomeric BR form (5-cis-26-cis-BR), whereas the presence of 9-cis-BR and 13-cis-BR was reduced [68]. Dehydrated derivatives of BR, monoanhydrobacterioruberin, bisanhydrobacterioruberin, and trisanhydrobacterioruberin and their isomers are also found in *Haloarchaea* extracts.

The C-50 BR ((2S,20S)-2,2′-bis-(3-hydroxy-3-methylbutyl)-3,4,3′,4′-tetrahydro-1,2,1′,2′-tetrahydro-, -carotene-1,1′-diol) along with its derivatives (Figure 7) is a red pigment with 13 C double bonds and four terminal hydroxyl groups, which constitutes 51–81% of the identified carotenoids from *Haloarchaeas* [65,66,68,132,133]. BR is produced by most *Haloferacaceae* members such as *Halobacterium salinarium*, *H. mediterranei*, *Haloferax volcanii*, *H. cutirubrum*, and *Halorubrum tebenquichense* and in small amounts by some highly radioresistant bacteria such as *Rubrobacter radiotolerans* and psychrophiles such as *Arthrobacter agilis* and *A. bussei* (in these bacteria, BR is a fatty acid-independent mechanism for regulating membrane fluidity; as a result of bacterial cold adaptation, BR increases the membrane fluidity and cell resistance to freeze–thaw stress [134,135]). The content of BR in the biomass is used to monitor the density of halophilic archaeal communities in halophilic environments.

In addition to supporting the archaeal ion pump rhodopsin, BR increases archaea membrane rigidity [137] and provides protection against UV light [138]. The presence of BR in the archaea membrane increases its hydrophobicity and minimizes intracellular water loss but allows oxygen molecules to pass through the cell membrane. Therefore, BR stabilizes archaeal cells under high osmotic and oxidative stresses [132,139].

The redness of flamingos is linked to the presence of carotenoids from halophilic archaeas inhabiting salt lakes and ponds where these birds nest [140]. Living halophilic archaea have been found in their feathers, and BR has been identified in the feathers’ structures. Halophilic archaeas are part of the flamingo’s diet; consequently, mainly BR is ingested, metabolized, and further assimilated [141]. Halophilic archaea are also a source of feed for metazoans thriving in the salt. *Artemia,* for example, survive assimilating nutrients from a halophilic archaea-based mono diet [142]. Feed containing *Haloferax volcani* and *Halorubrum* has improved the biomass production of *Artemias* and increased their antioxidant production [143,144], with BR being the major contributor to such a positive effect. Recently, haloarchaea were also validated as components of the human gut microbiome [145].

The relative amount of BR and the type of isomer depend on the archaea species and on the culture condition (namely, NaCl concentration, source of C, pO_2_, etc.). *All*-*trans* BR has a membrane-spanning (hydrophobic part: 36 Å [137]), fully unsaturated isoprenoid chain [146], and the terminal OH groups interact with hydrophilic headgroups of polar lipids via H bonds, which allows a single BR molecule to connect the inner and outer leaflet of the membrane bilayer [147]. A recent work reports that BR extracted from *H. tebenquichense* (acetonic extract) inserts into the bilayer of archaeosomes (vesicles made of polar diethers archaeolipids extracted from *H. tebenquichense*), slightly increasing its thickness, modifying the positions of the archaeolipids polar heads, and perturbing the longitudinal polyisoprenoid axe [55]. The monolayer-like BR is known also to stabilize the membrane of haloarchaea [137], possibly functioning as analogue to the membrane-spanning archaeal tetraethers [148], absent in halophilic archaea.

## 7. AST and BR Production and Extraction

Several companies located in different parts of the world (Israel, USA, China, India, Iceland, and Chile) market AST from *H. pluvialis* [149]. The production of AST at a competitive price is difficult because of the high costs involved in upstream and downstream processes of the *H. pluvialis* cultivation and AST extraction [150]. Achieving a more profitable production of a bioactive such as AST is a central economical question. In such a scenario, several reasons justify the extreme biotechnological interest on halophilic microorganisms. First, halophilic xanthophylls have evolved to efficiently protect these organisms against severe irradiative aggression, high temperatures (up to 50 °C in summer), and dehydration, conditions associated to an important oxidative stress [151]. Second, the procedures for pigment extraction and purification of xanthophylls seem to be simpler than those from other sources. Compared with plants or freshwater algae, a reduction in NaCl concentration is sufficient to induce the cell lysis, and carotenoid extraction could be conducted directly from the cells without any mechanical operation [152]. Third, from an industrial large-scale production, the risk of contamination of the halophilic culture with other microorganisms is reduced due to the high-salinity conditions used in their culture media. Halophilic archaea are of major biotechnological relevance since they can be cultured under non-sterile conditions employing cheap feedstocks that may be toxic to other microorganisms, significantly reducing the cultivation costs [152,153,154,155]. The simpler growth conditions of halophilic archaea constitute a remarkable advantage even compared with other archaea genera living in extreme hot, cold, acidic, or anaerobic environments (whose industrial provision of biomaterials is limited by their extreme life requirements, their slow growth rate, and their low production yield). In addition, halophilic archaea do not require the use of heterologous systems as production platforms to increase low yields of products [156,157]. The other side of halophilic cultures is the need for massive amounts of salt, a fact that is linked to a subsequent need for desalinization and culture media recycling [158,159].

Aiming to objectively analyze their biotechnological potentialities, the biomaterials extracted from archaea are classified within a scale of nine biotechnological readiness levels (BTRL), where each level is linked to a determined work achievement [155]. Currently, the only commercially available products from archaeas are extracted from halophilic archaea: BR and squalene (two non-polar archaeolipids), bacteriorhodopsin (membrane protein), and di-/tetraether-lipids [160,161]. These products do not exceed the BTRL 3, denoting that none of them is produced in industrial amounts despite being commercialized (by a recently created small German company named Halotek, which markets an extract rich in BR); their demand is satisfied by selling tiny amounts at very high prices.

## 8. Functional Foods vs. Nanomedicines?

In recent times the boundaries between the food and pharmaceutical sectors have blurred; the confusion regarding carotenoids’ status as drugs or as foods will be briefly discussed.

### 8.1. Few Carotenoids Are Regarded as Drugs

Most carotenoids are manufactured as foods or food components. Remarkably, from the more than 1000 carotenoids described to date, very few have shown therapeutic activity, are regarded as drugs (defined as “a substance (other than food) intended to affect the structure or any function of the body”, “a substance recognized by an official pharmacopoeia or formulary”, or “a substance intended for use in the diagnosis, cure, mitigation, treatment, or prevention of disease” [162]), and are manufactured under pharmaceutical regulations. The shortlist of carotenoid-based drugs includes vitamin A palmitate and retinoids (all-trans and 9’cis isomers of retinoic acid produced from retinol, 3,4- didehydroretinol, and retinal) [8,163].

### 8.2. Most Carotenoids Are Regarded as Food

Carotenoids are natural products regarded as bioactive compounds. Bioactive compounds are molecules usually found in tiny amounts as secondary metabolites produced by marine and halophilic microorganisms, yeast, algae, fungi, plants, and certain foods (such as fruits, vegetables, nuts, oils, and whole grains), having actions in the body that may promote good health, in the prevention of chronic diseases [32,164]. Of note, the vocabulary used in the vast field of natural products includes many terms that are not rigorously defined. An example is “nutraceutical” (a term launched in 1989 resulting from merging the words “nutrition” and “pharmaceutical”), coined by the industry. Nutraceuticals are said to be “a food (or a part of food) that provides medical or health benefits, including the prevention and/or treatment of a disease” [165,166]. Although not specifically defined by law, these products are regulated by the US Food and Drug Administration (FDA) under the authority of the Federal Food, Drug, and Cosmetic Act.

Bioactive compounds are part of functional foods (foods rich in substances regarded as effective against diseases) [167], which offer advantages beyond the basic nutritional functions by exhibiting physiological benefits and reducing the risk of chronic diseases [168,169]. Different from dietary supplements (intended to supplement the diet with one or more dietary ingredients, e.g., vitamins, minerals, amino acids, herbs, or other botanicals) [168,170], functional foods must show a beneficial effect in the normally consumed amount and cannot be commercialized as pills or capsules [171]. Advertisements and academic articles on functional food nutraceuticals suggest these products may act as drugs, that is, they suggest these foods may exert a therapeutic effect.

From a regulatory point of view, drugs and foods differ in their claims, that is, their intended use and labels. Drugs, for instance, must be registered by the FDA in the United States and by the European Medicinal Agency in Europe, before they can be sold or marketed, ensuring quality, safety, and efficacy, with the additional goal of minimizing side effects [172]. Foods (nutraceuticals/functional foods) or dietary supplements, instead, do not require this approval. Supplement companies are responsible for having evidence that their products are safe and that the label claims are truthful and not misleading. Foods are expected to comply with Current Good Manufacturing Practices—these outline facility standards, employee practices, and sanitation requirements, to ensure that the product is produced in a safe manner. Labeling standards for dietary supplements are grouped together with those for foods and regulated under the Food Drug and Cosmetic Act and the Dietary Supplement Health and Education Act (DSHEA) in the United States and the European Food Safety Authority in Europe [170].

Clearly, neither dietary supplements not functional foods are drugs. Both, however, are deemed an interphase between nutrition and pharma. Traditionally, drugs have been used to cure diseases or to alleviate the symptoms of disease. Nutrition, on the other hand, is largely meant to prevent diseases by providing the body with the optimal balance of macronutrients and micronutrients needed for good health. Due to the rising understanding of illness, drugs are now increasingly being used to lower risk factors and thereby prevent chronic diseases [173]. Currently, these factors are reduced by administering not only functional foods and dietary supplements but also drugs to “unhealthy” (though not diseased) patients. Functional foods, therefore, are not used to cure patients nor are they expected to have therapeutic effects, but they would reduce, in the best of cases, the risk factors for chronic diseases.

### 8.3. Protection of Carotenoids’ Labile Structure in Foods

It is worth to note that carotenoids’ structure is highly labile, with carotenes being in general more prone to thermal degradation than xanthophylls [174,175]. Their unsaturated backbone, for instance, is extremely sensitive to oxidation, hydrolysis, isomerization, and degradation mediated by heat, light, oxygen, catalysts, and other insults suffered during extraction and storage [174]. In addition, carotenoids are poorly available from natural sources, varying from 5 to 30%, in comparison with other food phytochemicals [176,177,178]. To protect their structure and increase their bioavailability, carotenoids in functional foods are loaded into particulate material such as liposomes, micelles, lipid nanoparticles, polymeric microparticles and nanoparticles, or nanocapsules [179,180,181,182]. Their controlled release reduces their lability and increases their bioaccessibility and bioavailability. Carotenoids are thus protected against physical–chemical and enzymatic aggressions; their shelf life is increased, as well as their stability along the gastrointestinal tract upon ingestion.

However, the introduction of the terms “liposomes, micelles, lipid nanoparticles, polymeric microparticles and nanoparticles, and nanocapsules” to the field of functional foods has brought confusion. Such terms used to make reference to “controlled release systems” in pharmaceutical nanotechnology [183] are also synonyms of “nanomedicines”, a new, sophisticated type of drugs. Liposomal nanomedicines, for instance, are designed and prepared in different conditions and expected to play a different role compared with liposomes in functional foods [184].

### 8.4. Characteristic Features of Nanomedicines

**Figure 8:**
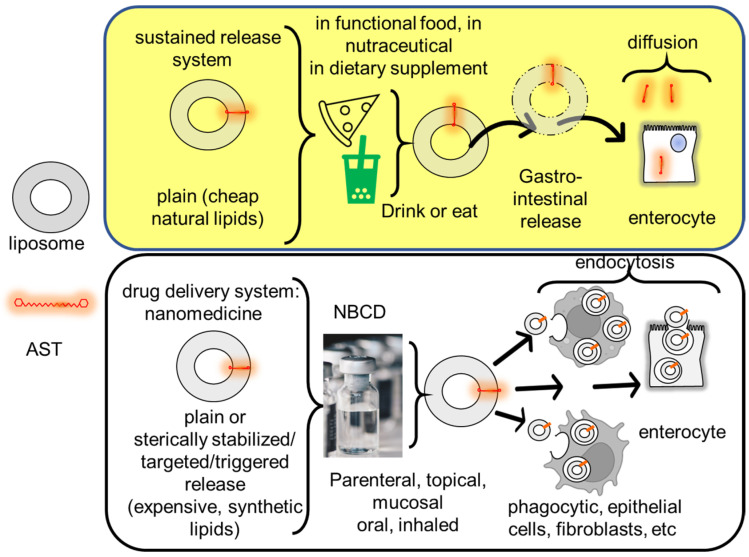
Main structural, administration routes and pharmacodynamic differences between liposomal AST as a sustained release system in functional foods and liposomal AST as a nanomedicine. NBCD: non-biological complex drug.

Nanomedicines consist of nanostructures loaded with different types of molecules; the first nanomedicine launched to the pharmaceutical market in 1995 was pegylated liposomal doxorubicin (Doxyl/Caelyx) [185]. Although supramolecular structures in nature, nanomedicines are considered single active pharmaceutical principles, and their structure cannot be fully characterized employing physicochemical methods, depends on its manufacturing process, and receives the name of “non-biological complex drugs” [186,187]. From their arrival to the pharmaceutical market until today, nanomedicines have been mainly used as antitumor agents [188]. Nanomedicines are specifically designed to be administered by diverse routes (not only oral or topical but also inhalation and mostly parenteral endovenous) and tailored to provide a given PK, BD, and PD of the loaded molecule [189]. The structure of intravenously administered nanomedicines is specially designed to retain loaded molecules against dilution after injection (and not to act as slow-release depots). Molecules (usually very toxic cytostats) formulated as nanomedicines remain confined to the vascular compartment, their access to healthy tissues being prevented. In addition, the relative control of the BD, provided by nanoparticle pegylation or small size and rigid nanostructure (such as that of liposomal vincristine sulfate, Marqibo), are responsible for its reduced clearance and long circulation, facts that maximize the chances of extravasation in solid tumors exhibiting permeable microenvironments [190]. Examples of these nanomedicines are liposomal pegylated doxorubicin (Doxyl, Caelix) and liposomal daunorubicin (Daunoxome). The main function of these classical nanomedicines was to reduce the loaded molecule’s toxicity, although their efficiency was comparable to that of the free loaded molecules. Only the latest generation nanomedicines, from albumin-bound nanoparticles (n-ab technology) for taxane delivery (Abraxane) to Cynviloq/Genexol-PM/Apealea, have shown increased efficiency thanks to their endocytic capture by target cells in tissues or transcytosis [191] toward epithelial cells, macrophages, or tumor cells [192]. In certain cases, the synchronous delivery of antitumor APIs in a certain proportion is achieved (Vyxeos) [193]. Similar scenarios arise with other types of API, such as the antifungal amphotericin B, which, when formulated as a nanomedicine, reduces its toxicity by preventing its access to the kidneys and redirecting its biodistribution to the liver [194]. Remarkably, the changes introduced in PK, BD, and PD depend strictly on the structure of the nanoparticulate material, which therefore needs to be carefully designed according to a rational plan (a strategy known as quality by design) [195]. In addition to fulfilling the claims of a drug, nanomedicines must be manufactured according to good pharmaceutical laboratory and manufacturing practices to ensure sterility and pyrogenous absence [196] (Figure 8). Despite there being no published specific guidance for nanomedicine, in 2017, the FDA produced draft guidance on drug products, including biological products, that contain nanomaterials, recommending evaluating each product on a case-by-case basis [197].

The reports on the performance of AST and BR-based nanomedicines will be analyzed in the next section; the structural features of nanomedicines, doses, and models are described in Table 2.

## 9. AST and BR-Based Nanomedicines

### 9.1. Nanomedicines for Oral Delivery of AST and BR

#### 9.1.1. Nanomedicines to Treat Inflammatory Bowel Diseases (IBDs)

IBDs, including Crohn’s disease and ulcerative colitis, are relapsing disorders of the gastrointestinal tract (GIT) with no cure, characterized by chronic inflammation and epithelial injury induced by the uncontrolled activation of the mucosal immune system [214]. OS is primarily responsible for IBD pathophysiology [215,216]. The ROS in the intestinal mucosa of IBD patients is 10- to 100-fold higher than that in healthy mucosa [217]. Whereas the co-administration of anti-inflammatory drugs and antioxidants has shown clinical benefits in IBD patients [218,219,220], no specific antioxidant treatment against IBD is currently available. Successful oral treatments depend on maintaining the drug’s structures along the gastrointestinal transit and on the feasibility of macrophage and dendritic cell targeting. Both AST and BR suffer gastrointestinal degradation. Targeting macrophages with AST or BR loaded nanoparticles aimed to protect them along the gastrointestinal transit could make IBD treatments more effective than conventional therapies.

AST loaded into caseinate microparticles covered with chitosan- triphenylphosphonium bromide (TPP, a well-known mitochondria-targeted moiety due to its high lipophilicity and stable positive charge [221]) and sodium alginate (CC-AST) were prepared by the electrostatic self-assembly method [198]. The sodium alginate (a natural polysaccharide containing a large number of carboxyl groups) cover provides pH-responsiveness to microparticles, which in the stomach are protonated and then are agglomerated, protecting thus the AST in the core. Upon reaching the intestine under alkaline conditions, the alginate is deprotonated, and the microparticles acquire negative charge and are redispersed. In vitro studies showed that CC-AST are internalized by RAW264.7 macrophages, accumulate in mitochondria, and inhibit ROS and mitochondrial membrane depolarization in LPS-induced macrophages. In a murine model of colitis, the previous and during-induction administration of CC-AST relieves colitis and significantly inhibits the expression of the inflammatory markers IL-1β, IL-6, TNF-α, cyclooxygenase-2, myeloperoxidase (MPO), iNOS, and NO in a more potent way than free AST. In addition, CC-AST protects the integrity of the colon tissue structure, maintaining the expression of the tight junction protein zonula occludens-1. Two dominant phyla, *Firmicutes* and *Bacteroides*, represent more than 90% of the intestinal commensal microbes of the human gut microbiota. The balance between *Firmicutes* and *Bacteroides* is related to host health: in the murine colitis model the abundance of *Bacteroides* is increased with the concomitant decreased ratio of *Firmicutes/Bacteroides.* CC-AST increases the relative abundance of *Firmicutes; Lactobacillaceae*, on the other hand, improves the intestinal environment, relieving inflammation.

The same authors have recently reported the preparation of ultrasonic assisted self-assembled Np, where AST was loaded into ROS-triggered self-disintegrating mitochondrial-targeted Nps made of poly (propylene sulphide) (PPS) and Rhodamine 123 (RD) covalently modified with sodium alginate [200]. PPS is a hydrophobic ROS-responsive functional group that can be converted from hydrophobic sulphide groups to hydrophilic sulfoxide/sulfone groups under ROS stimulation. RD, on the other hand, was used as a targeted ligand to mitochondria. The combination of PPS with sodium alginate at a 3:1 *w*/*w* ratio was required for the self-association of Nps. It was observed that the protonation of sodium alginate in simulated gastric fluid induced Nps aggregation with further deprotonation in simulated intestinal fluid, swelling, and AST release; the incubation with H_2_O_2_ instead disintegrated the Nps. The Nps accumulate in mitochondria, inhibit ROS, and protect against mitochondrial membrane depolarization in LPS-induced Raw264.7. The previous and during-induction administration of Np relieves the severity of colitis, protects the integrity of colon tissue, and restores the expression of ZO-1 and occludin (while free AST and void Nps do not). The abundance of *Lactobacillus* and *Lachnospiraceae* and the *Firmicutes/Bacteroides* ratio of gut microbiota are significantly improved.

In a similar approach, AST was loaded into Np having a core made of TPP-modified whey protein isolate-dextran conjugate (for mitochondrial targeting) and covered with hyaluronic acid (HA) modified with lipoic acid for macrophage targeting and a GSH-stimulated release feature (HL-TW-AST) [199]. Whereas HA has a strong affinity by cluster of differentiation protein 44 (CD44), overexpressed on the surface of activated macrophages in colitis tissues, the disulphide bonds of lipoic acid are reduced in the presence of high intracellular GSH in inflamed cells, releasing the Nps *core*. The lipoic acid–HA coating protects from early AST release in the stomach; macrophage and mitochondria targeting were shown in vitro on Raw264.7 cells. On LPS-induced macrophages, HL-TW-AST reduces ROS; reconstitutes to normal levels the mitochondrial membrane potential; increases the CAT, SOD, and GSH levels; reduces the iNOS, NO, TNF-α, IL-1β, and IL-6 levels; and increases the anti-inflammatory IL-10 cytokine levels. In murine colitis models, the previous and during-induction administration of HL-TW-AST markedly alleviates clinical symptoms (body weight loss, colon length, and spleen weight) and inflammation (decreases the level of malondialdehyde (MDA, a stable metabolite of lipid peroxidation); increases the levels of CAT and GSH; reduces MPO, iNOS, TNF-α, IL-1β, and IL-6; and increases IL-10 levels) more extensively than free AST. The anti-inflammatory effects of HL-TW-AST are mediated by the modulation of TLR4/MyD88/NF-κB signaling pathway. The HL-TW-AST administration is also shown to improve the composition of gut microbiota and the production of short-chain fatty acid.

A simpler approach was recently launched, where an o/w emulsion of AST in olive oil and soy lecithin was encapsulated in alginate microspheres by high-pressure spraying and ionic gelation with CaCl_2_ [201]. In vitro, the protection of AST along the GIT was observed, as well as its release in the colon thanks to the degradation of alginate by the gut microbiota. The microparticles increased the colon length, increased the liver weight, and reduced the spleen weight increase in a murine model of colitis. In the colon tissue, the microparticles are observed to reduce the levels of IL-6 and IL-1β, MPO, and iNOS and increase the levels of IL-10, ZO-1, occluding, SOD, and GPx, accompanied by increased *Firmicutes*/*Bacteroidetes* ratio.

Scavenger receptors class A (SR-A1) are involved in the innate immune response in intestinal inflammation [222]. SR-A1 negatively regulates NF-κB signaling and stimulates the production of reparative cytokines, shifting macrophage phenotypes from pro-inflammatory (M1) to anti-inflammatory (M2) [223]. Recently, nanostructured archaeolipid carriers (NACs) were loaded with BR plus dexamethasone (NAC-Dex). The NAC consisted of a compritol and BR core, covered by a shell of polar archaeolipids (PA) extracted from the halophilic archaea *Halorubrum tebenquichense* and Tween 80 [53]. The shell provides macrophage targeting because of its high content of 2,3-di-O-phytanyl-snglycero-1-phospho-(3′-sn-glycerol-1′-methylphosphate) (PGPMe), a ligand for SRA1 [224]. The shell provides also structural endurance since the PA (mixture of the *sn* 2,3 ether linked phytanyl saturated archaeolipids) is resistant to hydrolysis, oxidation, and stereospecific phospholipases [225]. NAC-Dex was observed to display high anti-inflammatory and antioxidant activities on a gut inflammation model made of Caco-2 cells and LPS-stimulated THP-1-derived macrophages, by reducing TNF-α and IL-8 release and ROS production. NAC-Dex also reverses the morphological changes induced by inflammation (normal microvilli, well-defined tight junctions, desmosomes, interdigitations, and F-actin filaments) and increases the transepithelial electrical resistance, partly reconstituting the barrier function. After in vitro gastrointestinal digestion, NACs retain their size and structure, while important, the anti-inflammatory activity of NAC-Dex remains intact, indicating the high structural resistance of Nps prepared with lipids extracted from halophilic archaebacteria.

#### 9.1.2. Nanomedicines to Treat Liver Damage

In a model of liver damage, orally administered liposomal AST before an LPS challenge was used to decrease and normalize the serum levels of glutamate-pyruvate transaminase, blood urea nitrogen (BUN), creatinine, and glutamate-oxaloacetate transaminase to levels comparable to those induced by the antioxidant N-acetylcysteine, more efficiently than free AST [209]. Liposomal AST also reduced the serum levels of NO, IL-6, and TNF-α more efficiently than N-acetylcysteine and free AST. In addition, the hepatic levels of MDA, SOD, and GPx were restored, and those of CAT were partially alleviated, while N-acetylcysteine and free AST provided moderate relief. The increased levels of iNOS and nuclear NF-κβ induced by LPS, were also decreased more efficiently than N-acetylcysteine and free AST. The authors suggest that liposomes increase the AST bioavailability, although neither the gastrointestinal stability of liposomal AST nor its biodistribution are reported. Moreover, a potential mechanism by which the AST bioavailability is increased is not provided; apparently, the AST bioavailability was increased despite the poor structural stability of liposomes in the GIT.

Recently, AST loaded into lactobionic acid (LA, targeting asialoglycoprotein receptors on hepatocytes) modified hydroxypropyl-β-cyclodextrin (AST-LA-CD) was administered for liver-targeting [210]. AST-LA-CD released 12% and 25.6% of AST after 2 h in simulated gastric fluids and 4 h in simulated intestinal fluids, respectively. In vitro, AST-LA-CD showed increased cellular uptake and prevented mitochondrial depolarization and ROS induced by H_2_O_2_, compared with non-targeted CD and free AST. After oral administration of Nile Red labeled-LA-CD (where AST was replaced with the hydrophobic fluorescent dye Nile Red, a molecule used to track its biodistribution) a higher fluorescence in liver was shown compared with non-targeted CD. This result could suggest that the LA-HD nanocarriers had stronger liver-targeted ability as compared with HD nanocarriers. However, free absorption of the dye could not be discarded given that Nile Red was not covalently attached to LA-CD. Therefore, no rigorous evidence showing cyclodextrin absorption to gain blood circulation is provided.

#### 9.1.3. Nanomedicines to Treat Inherited Retinal Degeneration

Retinal degeneration is a heterogeneous group of retinopathies affecting the outer layers of the retina, damaging the photoreceptor layers and the pigmentary epithelia, and perturbing the visual field, causing night blindness and altering color perception. If the molecular mechanism of the disease is still unknown and no specific treatment is available, the OS is known to be involved in the apoptosis of photoreceptor cells. Orally administered polysorbate 20 micelles loaded with AST (AST micelles) improved the architecture and functionality of the retina in a chemically induced mouse retinal degeneration model [206]. The AST micelles preserved photoreceptor responsiveness and inhibited photoreceptor loss in the degenerative retina, corroborated by electroretinography results and behavior tests, more efficiently than lutein (which is known to access the retina because of its ability to cross the blood–retinal barrier). Unfortunately, the work did not test the activity of free AST. The AST micelles significantly reduce the mRNA level of caspase-3 (mediator of photoreceptor apoptosis) and Bax and increase the mRNA level of the anti-apoptotic Bcl-2, showing anti-apoptotic effect. In addition, the MDA and 8-OHdG levels are reduced and the SOD and Mn-SOD levels in the retina are increased to a higher extent than those induced by lutein. Whereas AST PK and BD of micellar AST were not determined, this, together with the oral administration of liposomal AST, is another example of increased AST bioavailability mediated by nanomedicines.

### 9.2. Nanomedicines for Topical Delivery of AST and BR

#### 9.2.1. Nanomedicines to Treat Atopic Dermatitis (AD) and Psoriasis (PS)

OS and inflammation are involved in the pathogenesis and complications of cutaneous inflammatory diseases, such as AD and PS [226]. Topical treatments are ideal for direct action on the diseased cell: the first-pass metabolism and further systemic effects are avoided, they can be chronically applied, and the patient’s adhesion is good. However, AST and BR, in addition to being sensitive to oxidation induced by light and heat, do not permeate across the stratum corneum (SC) to enter the viable epidermis.

The AD is a chronic inflammatory affection of the skin, characterized by a massive release of proinflammatory cytokines and increased levels of IgE. In a recent work, despite not reporting skin permeation, liposomal AST was observed to decrease the severity of symptoms in a murine model induced by phthalic anhydride (displaying dermatitis, epidermal thickening, and mast cell infiltration) [202]. The topical treatment suppressed inflammatory mediators (iNOS and COX-2) and OS (reduced MDA levels and increased GSH, GPx, and HO-1) to a higher extent than free AST dissolved in acetone:olive oil 4:1 *v*/*v* ratio. Liposomal AST reduces STAT3 and p65 phosphorylation, inhibiting the activation of STAT3 and NF-kB. Remarkably, STAT3 is critical for regulating IgE levels, IgE-based allergen sensitization, and mast cell degranulation [227].

PS, on the other hand, is a multifactorial autoimmune skin disease, where OS plays a key role in promoting a vicious cycle between keratinocytes and immune cells. Recently, BR was loaded into NAC plus vitamin D3 (NAC-VD3) [71]. The combination of BR and VD3 in the NAC core produced a synergic antioxidant effect (measured by DPPH) and protected VD3 against thermal degradation. NAC-VD3 were extensively captured and displayed high anti-proliferative (65%), anti-inflammatory (IL-8 release), and antioxidant activities (ROS reduction) on a psoriatic model made of CaCl_2_ differentiated-imiquimod stimulated HaCaT cells and on lipopolysaccharide-induced THP-1 macrophages.

#### 9.2.2. Nanomedicines to Treat UV-Induced Skin Damage

UV irradiation generates ROS and induces skin damage such as inflammation, oxidative alteration of collagen, production of melanine, DNA alteration, and skin cancer in the long term. A topical pre-treatment with liposomal AST is effective to prevent morphological changes in a UV-induced mouse skin damage model, such as wrinkles and inflammation, better than free AST dissolved in DMSO [203]. Liposomal AST inhibits the increase in thickness of the *SC* induced by UV irradiation, preventing the decrease in the collagen amount below the *SC* layer. In addition, the combination of iontophoresis with cationic liposomal AST as a pre-treatment inhibits the UV-melanin production in the basal layer, indicating than liposomal AST prevents melanocyte damage from UV radiation. More recently, it was shown that the application of liposomal AST reverts the pathological changes induced by skin irradiation, such as the organization of collagen fibers and the dermis thickness, accompanied by decreased expression of Ki-67 (proliferation index related to cancer progression), MMP-13, and 8-OHdG (indicator of oxidative DNA damage) and increased SOD activity [204].

#### 9.2.3. Nanomedicines to Treat Dry Eye Disease (DED)

DED is a multifactorial disease, where the OS induced by the decreased volume of tears, their excessive evaporation, and hyperosmolarity play a key role. The daily application of topical drops containing liposomal AST on a murine model of DED prevented the increase in the fluorescein score (as a corneal damage measure) and the upregulation of age-related markers (p53, p21, and p16). The medication, however, did not increase the tears volume [205]. Liposomes displaying a small positive charge showed increased affinity by the cornea compared with neutral liposomes, efficiently ameliorating its deterioration, as a clinical symptom of DED, measured by superficial punctate keratopathy.

#### 9.2.4. Nanomedicines for Otoprotection

A frequent secondary effect of cisplatin chemotherapy is ototoxicity, which is produced by an excess of ROS. Aiming to increase the penetration through round window membranes, AST was loaded into lipid-polymer hybrid Nps made of a Peg-PLA and AST core covered by a shell of lipids (AST-Nps), prepared by an emulsion and evaporation technique [207]. The AST-Nps impair mitochondrial membrane potential reduction and avoid apoptosis (by suppressing the release of pro-apoptotic proteins, cleaving caspase 3/9 and cytochrome-c, and increasing the expression level of Bcl-2) induced by cisplatin on HEI-OC1 cells (House ear institute-organ of corti 1). AST-Nps but not free AST penetrate the round window membrane and maintain AST concentrations in the perilymph in the inner ear for 24 h after a single administration on guinea pigs. AST-Nps efficiently provide otoprotection to zebrafish hair cells against cisplatin and to guinea pigs exposed to cisplatin, especially in the higher and the ultrahigh frequencies.

### 9.3. Nanomedicines for Intra-Articular Delivery of AST

#### Nanomedicines to Treat Osteoarthritis (OA)

OA is a degradative disease of the cartilage caused by the mechanical wear and tear of the joints that mainly affects the hips and knees, and its incidence increases with age. OA involves the progressive loss of cartilage, remodeling of bone, inflammation, and deformation of the joint. Regardless of the causes, joint destruction is associated with the presence of proinflammatory cytokines including TNF-α, IL-1β, IL-6, immune cell subsets including macrophages, neutrophils, and activated synoviocytes [228,229] and expression of MMP that degrades the joint. In addition to inflammatory mediators, ROS and RNS play a key role in joint damage [230]. Macrophages and synoviocytes are the most abundant cells in the inflamed synovial and are fundamental for the progression of chronic inflammation and tissue destruction [229]. A ROS-responsive triblock copolymer (poly (ethylene glycol)-polythioketal-poly (ethylene glycol) (PEG-PTK-PEG)) through a simple and direct reaction between polythioketal (PTK) and m-PEG-acrylate, for AST delivery into the articulation was recently synthesized [212]. The polymer contains the lipophilic PTK segments in the middle of two hydrophilic PEG segments with a hydrophilic/lipophilic ratio of 31%, so that above the critical micellar concentration are formed micelles with a hydrophobic core where AST was encapsulated. In LPS-induced bone marrow-derived macrophages, PEG-PTK-PEG@AST micelles showed intracellular and extracellular ROS-scavenging effects higher than PEG-PTK-PEG and free AST, indicating a synergistic effect of PEG-PTK-PEG and loaded AST. Likewise, PEGPTK-PEG@AST micelles induce the transformation of the pro-inflammatory M1 phenotype provoked by LPS, to the anti-inflammatory M2 phenotype, and reverse the effect of LPS on IL-1β and TNF-α expression. In the OA rat model, PEG-PTK-PEG@AST micelles are gradually decomposed over time, remaining at day 7 in the OA tissues, after intra-articular injection. PEG-PTK-PEG@AST micelles show the best ROS-responsive scavenging ability and in vivo M1 transformation into the M2 phenotype. PEG-PTK-PEG@AST micelles demonstrate the most significant inhibitory effect on the expression of pro-inflammatory factors (MMP-2, IL-1β, TNF-α, and PGE-2) and on promoting the expression of marker proteins of cartilage anabolism (Col-2, aggrecan, and Sox-9), inhibiting the expression of cartilage catabolism (MMP-9 and MMP-13). The cartilage structure in the PEG-PTK-PEG@AST micelles group is better preserved.

### 9.4. Nanomedicines for Endovenous Delivery of AST

#### 9.4.1. Nanomedicines to Treat Liver Injury

The 3R,3′R isomer of AST resulted in the best isomer to be loaded together with capsaicin (CAP) (1:2 molar ratio) in liposomes bilayers to exert a synergic antioxidant effect [231]. Liposomal AST-CAP showed a protective effect in a murine model of liver injury upon intravenous administration, better than liposomal drugs loaded alone or combined treatment [208]. Liposomal AST-CAP significantly decreased the aspartate transaminase and alanine aminotransferase levels.

#### 9.4.2. Nanomedicines to Treat Diabetic Nephropathy

Diabetic nephropathy is a severe long-term complication from type 1 and 2 diabetes, which is presented as a decreased glomerular filtration rate, glomerulosclerosis, tubulointerstitial fibrosis, and renal tubular epithelial cell damage. The lesions are irreversible and lead to renal failure. OS is an important pathological mechanism of diabetic nephropathy, where the hyperglycemia promotes the generation of abundant ROS. The increased accumulation of proteins from the extracellular matrix (fibronectin and collagen IV) caused by ROS causes glomerulosclerosis and tubulointerstitial fibrosis [232,233]. Excessive ROS are aberrantly generated by renal-infiltrating or endogenous cells, then react with biomolecules to trigger kidney injury and renal dysfunction [234,235]. Aiming to reduce renal OS, AST was loaded in glucose-modified pegylated liposomes (AST-Glu-lipos) targeting glomerular mesangial cells that overexpressed glucose transporter 1 (GLUT1) on cell membrane [211]. AST-Glu-lipos retained AST in media with serum and pH 7.4, while a faster release at pH 5.5 was observed in a simulated lysosomal acidic environment. In vitro AST-Glu-lipos were more internalized, reducing ROS and apoptosis in Human Renal Mesangial Cells (HRMCs), especially high-glucose-induced HRMCs, to a higher extent than glucose lacking liposomes (AST-lipos) and free AST. In a diabetic rat model, while AST is filtered and excreted in urine, intravenously administered AST-lipos and AST-Glu-lipos were observed to accumulate in the renal cortex, including glomeruli and renal tubules, mainly mesangial cells, the latter being accumulated to a higher extent. AST-Glu-lipos increased the levels of SOD and Gpx and improved renal functionalism in terms of urine protein, serum creatinine, and BUN, as well as significantly improving renal pathological morphology.

### 9.5. Nanomedicines for Nose to Brain Delivery of AST

The OS is one of the key factors in the pathogenesis of neurodegenerative diseases such as Alzheimer’s and Parkinson’s. The neuroprotective activity of AST is reported by many studies. Aiming to increase its brain delivery, AST loaded into solid lipid nanoparticles (SLNs) prepared by the solvent displacement method was nasally administered [213]. The AST-SLNs were observed to protect from GSH consumption and from lipid peroxidation in PC-12 cells (from rat pheochromocytoma) upon H_2_O_2_-induced cellular injury, either as pre-treatment or as a post-treatment. After intranasally administering ^99m^Tc labeled AST-SLN to healthy animals, the amount of brain radioactivity compared with intravenous administration was increased.

## 10. Discussion

Historically, humankind has made use of the therapeutic activity of natural products [236]. The search for therapeutic activity in natural products, however, declined since the 90s due to three fundamental reasons: first, their heterogeneous and complex nature, which caused their efficient isolation and characterization to be difficult; second, their suboptimal efficacy, because of their poor absorption, distribution, metabolism, excretion, and toxicity (ADMET), which required the introduction of structural modifications of lead compounds either by chemical or semi-synthetic means or with genetically engineered organisms; and third, the difficulty in counting on a sustainable and economically viable supply of sufficient quantities of the compound. Recently, however, there has been a revival of industrial interest in seeking therapeutic applications for natural products, driven by new discovery techniques and analytics [237,238].

As described previously, xanthophylls are natural products that offer intense but labile antioxidant properties of pharmacological interest, whose exploitation may remain limited by the above factors. Their formulation as nanomedicines, however, may be key to solving the hurdles of using natural products as drugs. As shown in this review, nanomedicines controlled xanthophyll ADME without resorting to structural modifications or genetic engineering techniques. As a result, the activity of xanthophylls was magnified owing to their structural protection, which, in most cases, was followed by targeted delivery to selected cell groups.

Nanomedicine-based therapies allow changing the PK, BD, and PD of loaded molecules, as long as the right administration route is chosen. In this sense, the orally administered nanomedicines deserve a particular comment. Oral nanomedicines act on accessible epithelial targets in the gastrointestinal lumen; their structures remain intact until being endocytosed and delivered to the cellular interior. Due to their nanoparticulate nature, however, nanomedicines cannot cross epithelia upon oral administration (nor intact endothelium if intravenously administered). Neither the liver nor the eyes can be accessed by oral nanomedicines. Hence, the positive effects reported on liver damage and retinal degeneration probably resulted from enhanced drug bioavailability, where nanomedicines acted as a simple xanthophyll depot, but not from changes in xanthophylls’ PD. The first key step in enabling nanomedicine access to target cells, thus, is choosing the correct route of administration.

The remainder of the reported results resulted from the endocytosis of liposomes, micelles, and polymeric nanomedicines by accessible diseased tissues: nanomedicines were orally administered to be endocytosed by cells from the GIT surface; topically administered to treat the skin, eye surface, or ear; intraarticularly administered to be endocytosed by inflamed macrophages from the articulation; and intravenously administered to access deep organs. Some of them responded with phase transitions to changes in the environmental pH or were functionalized to target mitochondria. The structures of these nanomedicines were of greater complexity than those of nanoparticles or microparticles (liposomes, micelles included) in functional foods, which acted as depots for the slow release of carotenoids into the intestinal lumen. Different from diffusion, a process mediated by a molecular concentration gradient that is dependent on the administered dose, the endocytosis of nanomedicines occurs at the expense of cell energy. Switching the cell entry mechanism from diffusion to endocytosis is a way to modulate xanthophylls pharmacodynamics and is the second key to achieving antioxidant activity, even at low doses.

For instance, the most potent in vitro activators of the KEAP1/NRF2-mediated antioxidant response known to date [239] are the semi-synthetic derivatives of the oleanolic acid (pentacyclic triterpenoids bardoxolone methyl (also known as RTA 402) and omaveloxolone (RTA 408)) at nanomolar concentrations. In contrast, activating the KEAP1/NRF2-mediated antioxidant response requires a three-orders-higher concentration (micromolar) of free AST [240]. If formulated as nanomedicines, however, the antioxidant response should be triggered with doses lower than that of micromolar AST. Few endocytic events of low nanomedicine doses may be sufficient to increase the intracellular concentration of xanthophylls to the micromolar levels required to activate key metabolic pathways. Table 2 depicts the free AST/BR doses that in vivo lack activity but that triggered an intense antioxidant and therapeutic response when endocytosed as nanomedicines.

Different from corticosteroids, the resultant antiinflammation was mediated by mechanisms that would not disturb the mitosis of healthy cells. Nanomedicines revealed hidden beneficial activities of loaded molecules, potentially resulting in a fruitful strategy to create hundreds of novel molecular entities if applied to other carotenoids. The massive supply at relatively low costs of new powerful antioxidants, such as AST and BR, was granted by the use of microorganisms such as microalgae [241,242] or, even better, by halophilic or marine microorganisms from the Archaea domain as sources of AST and BR, respectively.

Carrying out rigorous structural characterizations, determining drug efficacy on relevant preclinical models and properly designed clinical trials, and constituting profitable products are among the main translational challenges that nanomedicines must overcome [243]. In view of that, how realistic is the idea of formulating xanthophylls in nanomedicines? Their structural characterization would be complex, not only because of the potential impurities present in xanthophylls extracts but also because the nanomedicine’s structure must maintain xanthophyll’s antioxidant activity throughout storage and gastrointestinal transit or across inflamed skin upon oral or topical administration, respectively, to target diseased tissues with minimal side effects. Most oral AST-based nanomedicines examined in this review, for instance, possess a complex constitution pursuing triggered responses to environmental pH or oxidative stress. Such complexity would cause their structural characterization to be even more difficult. On the other hand, the idea of using natural antioxidants for health benefits is relatively recent; there are nearly 100 current clinical trials employing free AST, most of them aimed to show the protection of tissues submitted to high oxidative stress on healthy subjects, not to screen for therapeutic effects [244]. AST and BR-based nanomedicines instead are intended to repair severely damaged tissues, and their efficacy was tested on preclinical models of chronic diseases. The clinical approaches used to treat chronic inflammatory diseases, however, are complex and involve the use of different types of medications, according to the disease stage and individual susceptibility. We observed that the experimental settings of preclinical models of IBD and psoriasis are oversimplified since only the performance of AST-based nanomedicines vs. that of free AST was screened, omitting comparisons with classical medication. Although probably not meaningful, toxicological studies are needed to explore the potential side effects of xanthophyll-based nanomedicines.

There are, on the other hand, unique beneficial aspects that xanthophyll-based nanomedicines may offer to the field of anti-inflammatory agents. The classical anti-inflammatory agents are corticosteroids and NSAIDs [245]. Corticosteroids have genomic and non-genomic effects that result in anti-inflammatory, immunosuppressive, and antimitotic activity [246]. None of them, however, can be administered by the oral route in chronical treatments since upon systemic biodistribution, they cause non-desired effects. Corticosteroids may cause hypothalamic–adrenal axis suppression (cushingoid syndrome, weight gain, or growth retardation in child); gastrointestinal (gastritis or bleeding), musculoskeletal (osteoporosis, osteonecrosis, or myopathies) damage; skin, ophthalmic, cardiovascular, and neuropsychiatric alterations; and up to lethal infections [247]. NSAIDs, molecules capable of inhibiting cyclooxygenases, may ultimately lead to important hemorrhages [248] and, recently, were found to have toxic effects on different groups of aquatic animals even at low, environmentally relevant concentrations [249]. In addition to direct reduction of oxidative stress through the scavenging of ROS, RNS, and ^1^O_2,_ the antioxidant activity of xanthophylls is exerted at multiple levels: inhibition of nuclear migration of NF-κβ, MAP kinase activation, and activation of the PI3K/Akt and KEAP/Nrf2 response. Xanthophylls reduce oxidation and associated inflammation by mechanisms other than those of corticosteroids, and importantly, their free forms lack not only therapeutic activity, but also toxicity.

## 11. Conclusions

Nanomedicines offer key opportunities to transform xanthophylls into therapeutic agent alternatives to chronic oral or topical corticosteroids or NSAIDs. Submitting severe inflammatory diseases to long-term treatments with oral (acting exclusively on the luminal side of the GIT) or topical (acting exclusively on the cells of inflamed epidermis) anti-inflammatory AST or BR-based nanomedicines, in addition to aiding higher patient compliance, would reduce the frequency of parenteral administration of biologicals and is a strategy worth being explored.

## Figures and Tables

**Table 1:** Characteristics and key properties of AST and BR.

Carotenoid/ Property		AST	BR
**Production**	Natural source	*H. pluvialis:* high cost and low production	Halophilic archaea
	Chemical synthesis	Low-cost labor Inexpensive chemicals	Not reported
**Production volume/market**		190 Tn 2021: USD 647 million	Not reported
**Isomers**	Optical	*H. pluvialis*: 3S, 3′S Synthetic: 3S,3′S: 3R,3′S: 3R,3′R 1:2:1 ratio	None
	Geometric	All-trans (all-E) and cis isomers (9-Z, 13-Z, 15-Z) *H. pluvialis:* 73% all-E-AST and 27% cis-AST	All-trans, cis isomers (5-Z, 9-Z, 13-Z), and double isomers (5-Z-26-Z, and 9-Z-26-Z)
**Esterification**		Yes	No
**Biological role**		Oxygenic photosynthesis (light harvesting) and photoprotection	Defense against osmotic stress and radiation [47] Structural support to rhodopsin complexes (a retinal protein-carotenoid complex) [48]
**Physicochemical role on membrane**		None	↑ membrane rigidity ↓ water permeability
**Antioxidant role**	^1^O_2_ quenching	800 times > coenzyme Q 6000 times > vitamin C 550 times > green tea catechins, 11 times > β-carotene [49], natural 50 times > synthetic	Not reported
	Free radical scavenger	65 times > vitamin C 50 times > vitamin E [50] Natural 20 times > synthetic [51]	DPPH (IC50) *Hfx. mediterranei* extract: 40–74 μg/mL, *Haloterrigena* sp. [52], *H. tebenquichense* [53], *Halorubrum* sp. *BS2* [54] extracts: 3–6 µg/mL Haloarchaeal strains from Atacama Desert [55]: 4.2–34.7 µg/mL
	Preventing lipid peroxidation	100–500 times > vitamin E	*H. tebenquichense* extract protected red cells against peroxyl radical-induced hemolysis (IC50: 1.9 μg/mL) [55]
**Toxicity**		NOAEL (no observed adverse effect levels) natural AST is 465 and 557 mg/kg/day in male and female rats, respectively. The repeated-dose oral toxicity in pregnant mice showed LD50 > 20 g/kg [56].	Up to 500 mg/kg/day for 14 days on Wistar rats, no observed adverse effects were registered.
**In vivo/in vitro activity**		In vivo animal models: anti-neurodegenerative diseases [57], hepatoprotective [58], anti-cardiovascular diseases [59], inhibited the development of COPD and acute lung injury [60], improve dyslipidemia and metabolic syndrome [61], anti-diabetic nephropathy [62], burn wounds healing [63], and immunostimulation [64] In vitro: anti-fibrotic, and bone disease healing	In vivo: not reported In vitro: antioxidant, anti-inflammatory [55]; antiviral and anti-cancer activity [65] Cholinesterase [66], cyclooxygenase-2 [67], α-glucosidase, α-amylase, and pancreatic lipase [68] inhibition Beneficial effects on sperm cell viability [69] Antimicrobial activity against pathogenic bacteria and fungi [54,70,71]
**Metabolic role**		In animals ↑ fecundity, growth rate, egg yolk volume and quantity, intensity of flesh color, and strengthening of immune responses [72] ↑ lipids and glucose metabolism [73].	Not reported
**Intracellular target**		Mitochondria	Not reported
**Signaling pathways**	Nrf2	↑ NADPH, GSH, and OS-responsive enzymes [74,75] in brain, heart, kidney, eyes, lungs, skin, and liver [76].	Not reported
	PI3K/Akt	↑ downstream signaling mediators, including mTOR, and Nrf2 [77].	Not reported
	NF-κB	↓ TNF-α, IL-6, IL-1β prostaglandin E, inducible nitric oxide synthase (iNOS), and COX-2, in macrophages and neutrophils, ↓ inflammation in vivo [78].	Not reported
	Others	↓ JAK/STAT-3, PPARγ, and p38 MAPKs [79]	Not reported
	Apoptosis	Proapoptotic and antiapoptotic Anti-ROS generated-apoptosis: blocks caspase 3 and 9, cytochrome c, p-ERK/ERK, and decrease the Bax/Bcl2 ratio [75,77].	Proapoptotic: induced caspase-mediated apoptosis and inhibit MMP-9 in cancer cells [80]
**Animal uses**		Aquaculture feed (synthetic)	Aquaculture feed (artemia)
**Human uses**		Food supplements (natural), nutraceutical (natural), cosmetic ingredient (synthetic)	Cosmetic ingredient
**Clinical trials**		Several trials with dietary AST Preventive effects against atherosclerosis [78]; neuroprotective against cognitive impairment [81]; improved visual acuity and retinal blood flow [82]; reduced the signs of skin aging [83].	Not reported

Abbreviations: IC50: 50% DPPH inhibition; ↑ increase; ↓ reduce.

**Table 2:** Target disease, administration route, and type of nanomedicine for AST and BR delivery.

Disease/Route of Administration	Carotenoid/ Source	Np Type, Composition, and Structural Features	Type Studies/Dose	Reference
IBD oral	AST crude (5% purity) from Shandong Wefirst Biotechnology Co., Ltd. (Shandong, China) Natural AST from *H. pluvialis*	Polymeric microparticles: caseinate, chitosan-TPP and sodium alginate 1.7 μm (10 mg AST) EE: 55% LC: 50 μg/mg Neutral ξ potential	Raw264.7 macrophages Murine DSS model 12.5 mg/kg/day 7 days of treatment, then 6 days DSS + treatment	[198]
IBD oral	AST crude (5% purity) from Shandong Wefirst Biotechnology Co., Ltd. (Weihai, Shandong, China) Natural AST from *H. pluvialis*	Polymeric Np: poly (propylene sulfide) and Rhodamine 123 covalently modified sodium alginate 260 nm EE: 69%; LC: 3.6 μg/mg	Raw264.7 macrophages Murine DSS model 1.25 mg/kg/day 11 days of treatment, then 6 days DSS + treatments	[199]
IBD oral	AST crude (10% purity) from Xi’an Realin Biotechnology Co., Ltd. (Xi’an, China) Natural AST from *H. pluvialis*	Polymeric Np: TPP-modified whey protein isolate-dextran conjugate, covered by lipoic acid-modified HA 380 nm; −31 mVξ potential EE: 81%; LC: 2.7%	Raw264.7 macrophages Murine DSS model 10 mg/kg/day 14 days of treatment, then 7 days DSS + treatment	[200]
IBD oral	AST (purity >95%) from Solarbio Life Sciences (Beijing, China). The origin is not specified. Synthetic.	Olive oil-lecithin o/w emulsion encapsulated in sodium alginate microparticles 0.5–3.2 μm EE: 87%	Murine DSS model 30 ppm DSS and Np treatments at the same time once a day for 9 weeks	[201]
IBD oral	BR extracted from *H. tebenquichense*	NAC Compritol, BR, polar archaeolipids, and Tween 80 (2: 2: 1.2: 3% *w*/*w*) 66 nm; −32 mV ξ potential	THP-1 derived macrophages, Caco-2 cells gut inflammation model 3.5.10^−4^ μg/mL	[53]
Atopic dermatitis topical	AST from GDE Co., Ltd. (Siheung, Republic of Korea). The origin is not specified. Synthetic.	Liposomes SPC 65 nm	PA-induced (three times a week for 4 weeks) AD on mice 0.2 mg Liposomal treatment 3 h after PA induction	[202]
Psoriasis topical	BR extracted from *H. tebenquichense*	NAC 70 nm; −39 mV ξ potential	CaCl_2_ differentiated HaCaT cells imiquimod stimulated psoriasis model 3.5.10^−4^ μg/mL	[71]
UV-induced skin damage topical	AST from Sigma-Aldrich (USA) *	Liposomes EPC and DOTAP/EPC/Chol (2:2:1) 326 nm; −2 mV ξ potential and 170 nm; 43 mV ξ potential, respectively	UV treatment on Hos:HR-1 hairless mice dorsal skin once a day for five consecutive days 18 μg	[203]
UV-induced skin damage topical	No information available	Liposomes	C57BL/6J mice UVB irradiated one time per day for the first five days, and one time every other day for the next nine days	[204]
Dry eye disease topical	AST from FUJIFILM Wako Pure Chemical Corporation (Japan). The origin is not specified (3S,3’S).	Liposomes EPC or EPC/DOTAP (18:2 molar ratio) 130 nm; −0.4 mV ξ potential and 85 nm; 9 mV ξ potential, respectively	Rat DED model 0.6 μg Six times a day for 13 days	[205]
Retinal degeneration oral	AST (>90%) from Xian Zelang Biotech, China. The origin is not specified. Synthetic.	Micelles Polysorbate 20 (Tween 20) 76 nm; −16.5 mV ξ potential	N-methyl-N-nitrosourea (MNU) retinal degeneration mouse model 100 mg/kg Eight times 6 h before and at 0, 6, 12, 24, 36, 48, and 72 h after MNU IP administration	[206]
Otoprotection local administration	AST from Sigma-Aldrich (USA) *	Lipid–polymer hybrid Np (mPEG-PLA-DMPC) 91 nm; −10 mV ξ potential	Zebrafish and guinea pig exposure to cisplatin 5 μg	[207]
Liver injury endovenous	AST (3R, 3’R) from Sigma-Aldrich (USA) Synthetic	Liposomes EPC 114 nm	CCl_4_-induced liver injury rat model 298 μg/kg	[208]
Liver injury oral	AST (>99%) from the Fuji Chemical Industry Co., Ltd. (Toyama Prefecture, Japan) Natural AST from *H. pluvialis*	Liposomes SPC/Chol (4:1 *w*/*w*) 240 nm	LPS-induced liver injury rat model 2, 5, or 10 mg/kg/day Administered once a day 8 days before LPS IP challenge	[209]
Liver injury oral	AST (10% purity) was bought from Xi’an Realin Biotechnology Co., Ltd. Natural AST from *H. pluvialis*.	Hydroxypropyl-β-cyclodextrin 98 nm; EE: 74%	HepaRG cells Mice biodistribution	[210]
Diabetic nephropathy (DN) endovenous	AST from Innochem (Beijing, China). The origin is not specified. Synthetic.	Liposomes EPC/Chol/Glucose-PEG600-DSPE 95:20:5 molar ratio 120 nm; −31 mV ξ potential EE: 80%; LC: 6.8%	HRMCs cells 10 mg/kg DN rat model rats fed with high-sugar and high-fat fodder for 6 weeks followed by streptozocin injection	[211]
Osteoarthritis intra-articular injection	AST from Shanghai *Macklin* Biochemical Co., Ltd. (Shanghai, China). The origin is not specified. Synthetic.	Polymeric micelles poly (ethylene glycol)-polythioketal-poly (ethylene glycol) EE: 94%; LC: 9.4%	Bone marrow-derived macrophages 37 μg Rat OA model (intra-articular injection of monosodium iodoacetate into each left knee) Single injection 3 days after OA induction	[212]
Nose to brain	AST from Algaltech, Israel Natural AST from *H. pluvialis*	SLN stearic acid (50 mg), % AST (6.11%) and poloxamer 188: lecithin (1: 6) 206 nm; EE: 77%; LC: 47%	PC-12 cells 4 mg/kg Rat biodistribution	[213]

Abbreviations: AD: atopic dermatitis; Chol: cholesterol; DED: dry eye disease; DMPC: 1,2-Dimyristoyl-sn-glycero-3-phosphocholine; DN: diabetic nephropathy; DOTAP: Dioleoyl-3-trimethylammonium propane; DSS: dextran sodium sulfate; EE: encapsulation efficiency; EPC: egg phosphatidylcholine; HA: hyaluronic acid; LC: loading capacity; LPS: lipopolysaccharide; NAC: nanostructured archaeolipid carrier; mPEG-PLA: methoxy (polyethylene glycol); Np: nanoparticles; OA: osteoarthritis; PA: phthalic anhydride; PS: psoriasis; SLN: solid lipid nanoparticles; SPC: soybean phosphatidylcholine; TPP: triphenylphosphonium bromide. * Sigma markets different forms of AST: all trans from Blakeslea trispora, (3*S*,3′*S,all-trans*) AST, esters from *H. pluvialis*, USP standard (isomers mix), 9-cis and 13-cis.

## Data Availability

Not applicable.

## Abbreviations

8-OHdG	8-Hydroxy-2’-deoxyguanosine
AD	atopic dermatitis
ADME	absorption, distribution, metabolism, excretion
ARE	Antioxidant Response Element
AST	astaxanthin
BD	biodistribution
BR	bacterioruberin
BTRL	biotechnological readiness levels
BUN	blood urea nitrogen
CAGR	compound annual growth rate
CAT	catalase
CAP	capsaicin
cdb	conjugated double bonds
Chol	cholesterol
COPD	chronic obstructive pulmonary disease
DED	dry eye disease
DMPC	1,2-Dimyristoyl-sn-glycero-3-phosphocholine
DMSO	dimethylsufoxide
DN	diabetic nephropathy
DOTAP	dioleoyl-3-trimethylammonium propane
DPPH	1,1-diphenyl-2-picrylhydrazyl
DSS	dextran sodium sulfate
EE	encapsulation efficiency
EPC	egg phosphatidylcholine
FDA	Food and Drug Administration
GIT	gastrointestinal tract
GPx	glutathione peroxidases
GSH	glutathione
GST	glutathione S-transferases
HA	hyaluronic acid
HEI-OC1	house ear institute organ of corti 1 cells
HO-1	heme oxygenase-1
HRMCs	Human Renal Mesangial Cells
IBD	inflammatory bowel disease
IC50	half inhibition concentration
IL	interleukin
JNK	c-Jun N-terminal kinase
Keap1	Kelch-like ECH- associated protein 1
LA	lactobionic acid
LC	loading capacity
LD50	lethal dose 50
LPS	lipopolysaccharide
MAPKs	mitogen-activated protein kinases
MDA	malondialdehyde
MMP	matrix metalloproteinases
mPEG-PLA	methoxy (polyethylene glycol)
MPO	myeloperoxidase
MRP	multidrug resistance-associated protein
NAC	nanostructured archaeolipid carriers
NF-κβ	Nuclear factor kappa-light-chain-enhancer of activated B cells
Np	nanoparticle
NQO1	dehydrogenase quinone 1
Nrf2	Nuclear factor erythroid-related factor 2
NSAID	nonsteroidal anti-inflammatory drug
OA	osteoarthritis
OS	oxidative stress
PA	phthalic anhydride
PA	polar archaeolipids
PD	pharmacodynamics
PEG	poly (ethylene glycol)
PGPMe	3′-sn-glycerol-1′-methylphosphate
PK	pharmacokinetics
PPS	poly (propylene sulphide)
Prxs	peroxiredoxins
PS	psoriasis
PTK	polythioketal
RD	Rhodamine 123
RNS	reactive nitrogen species
ROS	reactive oxygen species
SC	stratum corneum
SLN	solid lipid nanoparticles
SOD	superoxide dismutase
SPC	soybean phosphatidylcholine
SR-A1	Scavenger receptors class A
TNF-α	Tumor Necrosis Factor-alpha
TPP	triphenylphosphonium bromide
Trx	thioredoxin
TrxR	thioredoxin reductase
